# Understanding the Dry Eye Disease-Related Symptoms in South America: Prevalence and Associated Factors—A Systematic Review

**DOI:** 10.3390/jcm13206060

**Published:** 2024-10-11

**Authors:** Valentina Loaiza-Guevara, Camila Salazar-Santoliva, Alvaro J. Villota-Arevalo, Marjorie E. Acosta-Villas, Betty-Liliana Coral-Gaón, Jesús E. Afanador, Natalia Restrepo, Laurens L. Hernandez-Benitez, Wendy Rincón Hernández, Laura C. Caceres-Delgado, Juan S. Izquierdo-Condoy

**Affiliations:** 1 Facultad de Medicina, Fundación Universitaria Autónoma de las Americas, Pereira 660001, Colombia; 2 One Health Research Group, Faculty of Medicine, Universidad de las Americas, Quito 170137, Ecuador; 3 Facultad de Medicina, Universidad Tecnológica de Pereira, Pereira 660003, Colombia; 4 Facultad de Medicina, Pontificia Universidad Javeriana, Bogota 110231, Colombia; 5 Facultad de Medicina, Universidad de Pamplona, Cúcuta 540004, Colombia; 6 Facultad de Medicina, Universidad Libre, Barranquilla 080003, Colombia; 7 Facultad de Medicina, Fundación Universitaria San Martin, Bogotá 110311, Colombia; 8 Facultad de Medicina, Universidad Autónoma de Bucaramanga, Bucaramanga 080003, Colombia

**Keywords:** dry eye disease, prevalence, associated factors, South America

## Abstract

**Background/Objectives**: Dry eye disease is a leading cause of ophthalmologic consultations worldwide and can significantly impact quality of life. While global prevalence rates vary widely, data specific to South America are limited. This systematic review aims to describe and analyze the prevalence and associated factors of dry eye disease-related symptoms in South American populations. **Methods**: Following Preferred Reporting Items for Systematic Reviews and Meta-Analyses (PRISMA) guidelines, a systematic review was conducted using databases such as PubMed, Web of Science, Scopus, and LILACS. Primary studies in English and Spanish that examined the prevalence of dry eye disease-related symptoms in South American populations and its associated factors were included without date restrictions. Studies were screened and selected based on predefined inclusion and exclusion criteria, resulting in the final inclusion of 16 studies from six South American countries. **Results**: This review identified significant variability in the prevalence of dry eye disease-related symptoms in South American populations in the region, ranging from 4% to 77.5%, with a mean prevalence of 39.3%. Higher prevalence rates were observed among specific groups, such as university students (58.6%) and administrative workers (57.9%). Factors associated with dry eye disease-related symptoms in South American populations included female sex, older age, prolonged screen time, insufficient sleep, and medical conditions such as hypertension, connective tissue disorders, and the use of medications like antihypertensives and antidepressants. **Conclusions**: The prevalence of dry eye disease-related symptoms in South American populations is notably higher than global averages, highlighting regional challenges. This study emphasizes the need for standardized diagnostic tools and comprehensive epidemiological research across South America, particularly in underrepresented countries, to inform public health strategies tailored to the specific needs of these populations.

## 1. Introduction

Dry eye disease (DED) is a leading cause of ophthalmological consultations worldwide. This complex, multifactorial disorder disrupts tear film stability due to dysfunction of the lacrimal and meibomian glands, resulting in inadequate tear production [1]. Consequently, patients frequently experience symptoms such as burning, itching, and blurred vision, as well as signs such as ocular redness, all of which can significantly diminish their quality of life. Moreover, various factors—including underlying diseases, medications, and environmental conditions—can exacerbate the condition [2,3].

Prevalence rates of DED show considerable variability, ranging from 11.59% to 50%, with higher rates observed in Asian populations, particularly in China, Tibet, and Korea [4,5,6,7]. In contrast, the prevalence of DED in the Americas is less well-defined, with most data stemming from the United States, where it is estimated at approximately 8.1% [5]. Specific data regarding South America remain scarce, and recent systematic reviews have largely focused on Brazil and Chile [8]. Consequently, achieving a unified estimate of prevalence in the region is challenging, and the existing data are heterogeneous. These limitations can be attributed to difficulties in accessing specialized healthcare; despite the multitude of issues available, there is a pressing need for specialized ophthalmological care for diagnostic confirmation [9,10]. Additionally, many patients struggle to recognize DED, as its symptoms—such as eye irritation, itching, burning, and blurred vision—are easily confused with other eye conditions.

Clinically, DED is often perceived as a minor condition; however, it is one of the most common disorders encountered in ophthalmologic practice. Its impact extends beyond physical discomfort, significantly affecting patients’ quality of life, work productivity, and social interactions [11]. This issue is particularly concerning for vulnerable populations, such as the elderly and individuals with autoimmune diseases [12].

In recent years, the prolonged use of digital screens has emerged as a significant risk factor for the development and exacerbation of DED, attributable to factors such as reduced flicker frequency and extended exposure to screen light [13,14]. Despite the recognition of various risk factors for DED, a knowledge gap persists regarding their prevalence and impact, specifically within South American populations [15]. Often underestimated, DED has a substantial effect on the daily lives of millions of individuals [16].

Therefore, this systematic review aims to describe and analyze the prevalence of dry eye disease-related symptoms and their associated factors in South American populations.

## 2. Materials and Methods

### 2.1. Study Design

We conducted a systematic review following the Preferred Reporting Items for Systematic Reviews and Meta-Analyses (PRISMA) guidelines [17].

### 2.2. Search Strategies

An extensive bibliographic search was conducted in both English and Spanish to ensure a comprehensive review of the literature. We utilized key databases, including PubMed/Medline, Web of Science, Scopus, Google Scholar, Scielo, and Latin American and Caribbean Literature in Health Sciences (LILACS). Additionally, a snowball technique was employed to examine the reference lists of relevant articles, identifying any studies related to the prevalence of factors associated with dry eye disease in South American populations that may have been overlooked. The search targeted primary studies without date restrictions. The search targeted primary studies without date restrictions.

The search strategy included specific index terms, keywords, and Boolean operators: (“Dry eye” OR “Dry eye disease” OR “Dry eye syndrome”) AND (“use” OR “usage” OR “consume”) AND (“prevalence” OR “frequence”) AND (“risk factors” OR “associated factors”) AND (“South America” OR “ Suriname” OR “Guyana” OR “Colombia” OR “Venezuela” OR “Ecuador” OR “Brazil” OR “Bolivia” OR “Chile” OR “Argentina” OR “Uruguay” OR “Paraguay” OR “Peru”) in the title (TI) or abstract (AB).

### 2.3. Selection Criteria

Given the variability in diagnostic tools for DED and the lack of data specific to the South American region, this review aimed to include studies that utilized questionnaires for identifying or self-reporting symptoms related to DED, as well as studies employing clinical diagnostic tests for DED in South American populations. The specific inclusion and exclusion criteria for study selection are outlined below.

#### 2.3.1. Inclusion Criteria

Studies in English or Spanish.

Primary research examines the prevalence and factors associated with dry eye disease-related symptoms in South American populations.

#### 2.3.2. Exclusion Criteria

Studies focusing on populations outside South America.

Secondary research such as systematic reviews, narrative reviews, scoping reviews, and meta-analyses.

Non-original research like perspectives, commentaries, opinion articles, and editorial letters.

### 2.4. Selection of Studies

**Figure 1:**
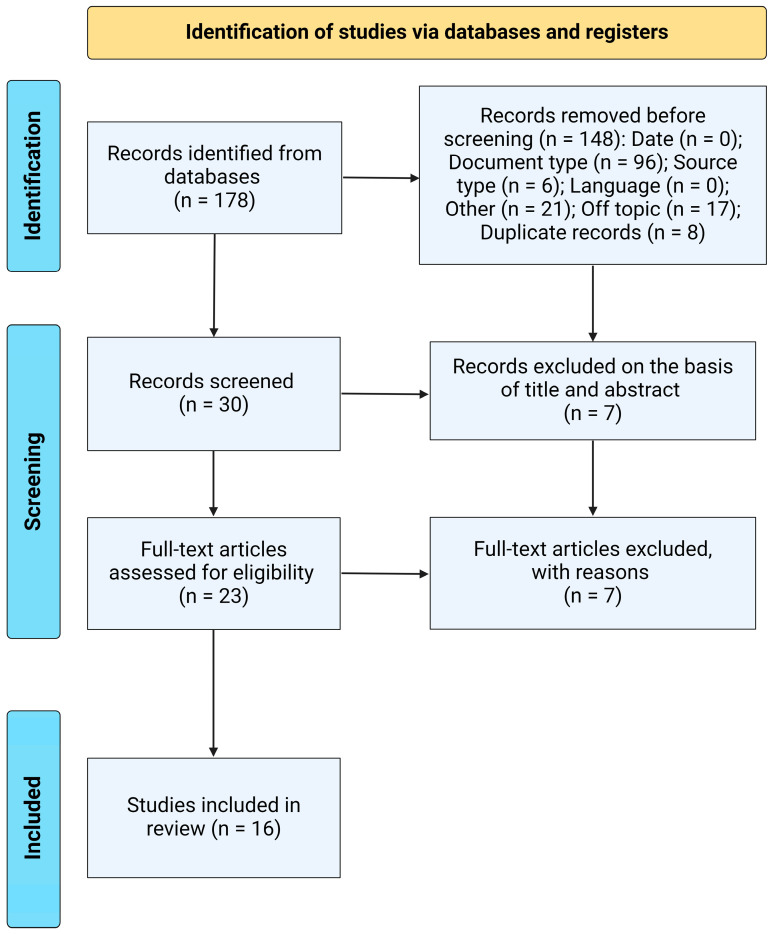
PRISMA flowchart illustrating the study selection process for this systematic review, detailing the number of studies screened, assessed for eligibility, and included in the review, with reasons for exclusions at each stage.

The initial search yielded 178 articles: 28 from PubMed/Medline, 14 from Web of Science, 18 from Scopus, 98 from Google Scholar, 8 from SciELO, and 12 from LILACS. During the first screening phase, 148 studies were excluded, primarily due to their document type (n = 96), such as review articles, editorial letters, opinion articles, and commentaries, with an additional 8 studies removed as duplicates. Of the remaining 30 articles, 7 studies were excluded due to limitations found in the title or abstract. Finally, the 23 eligible articles were reviewed in their entirety, and 16 studies were included in this research (Figure 1).

### 2.5. Bias Assessment

To minimize the risk of bias, two members of the research team (JIC, CS) independently performed the data extraction process at different times. Any discrepancies found during the data collection phase were resolved through discussion until consensus was reached among all members of the research team. This method was implemented to ensure the accuracy and reliability of our findings.

### 2.6. Data Synthesis

A comprehensive review was conducted of all manuscripts that met the established selection criteria. Information from these manuscripts was extracted, meticulously organized, and synthesized into descriptive tables and figures. This format was chosen to present our results in a clear and concise manner, facilitating reader understanding.

## 3. Results

Sixteen studies from 6 South American countries (Argentina, Brazil, Chile, Ecuador, Peru, and Venezuela) were included in this review, covering data from 2008 to 2023. Most studies (fourteen out of sixteen) employed a cross-sectional design, with sample sizes ranging from 60 to 10,812 participants (Table 1).

**Figure 2:**
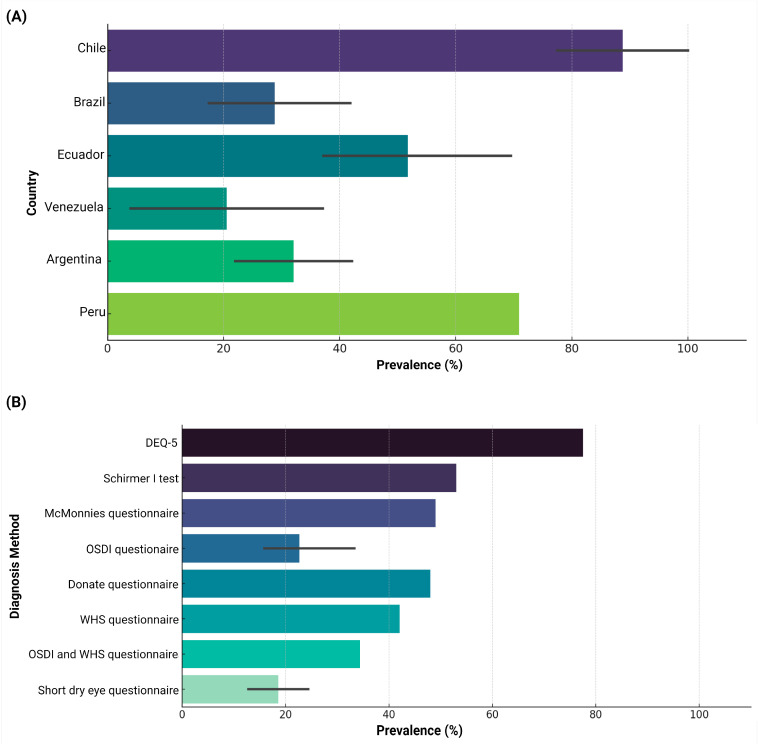
Prevalence of symptoms related to dry eye disease in South America. (**A**) Distribution of the prevalence of dry eye disease-related symptoms by country. (**B**) Distribution of the prevalence of symptoms related to dry eye disease according to the diagnostic method used.

These studies aimed to determine the prevalence of dry eye disease-related symptoms using various measures, including questionnaires and clinical diagnostic tests. The most frequently used questionnaires were the Ocular Surface Disease Index (OSDI) in five studies [21,24,26,29,31], the Women’s Health Study (WHS) dry eye questionnaire in two studies [28,29], and the Short Dry Eye Questionnaire in two studies [30,33]. The estimated prevalence of dry eye disease-related symptoms varied widely, from 4% [32] to 77.5% [18], reaching 100% in a validation study involving patients with clinically confirmed dry eye [27]. Excluding the 100% prevalence reported by Goya M. et al. [27], the overall average prevalence was 39.3% (Table 2 and Figure 2). Complementarily, although seven studies incorporated clinical diagnostic tests, only Holgado M. et al. [24] reported a prevalence of 27% as measured by OSDI. This figure is notably lower than those obtained through the lissamine green staining test (46%) and the precorneal rupture time test (52%) (Table 2). When considering the type of population studied, among the general population, the average prevalence was 23.0% [28,29,31,32,33], while higher rates were observed among administrative workers (57.9%) [20,22,25] and university students (58.6%) [18,26,29]. Prevalence according to specific etiologies varied as well, with computer vision syndrome-associated dry eye disease-related symptoms prevalence at 53.5% [18,20,24,25,26], significantly higher than the general dry eye disease-related symptoms prevalence of 32.8% [21,22,23,28,29,30,31,32,33] (Table 2).

**Figure 3:**
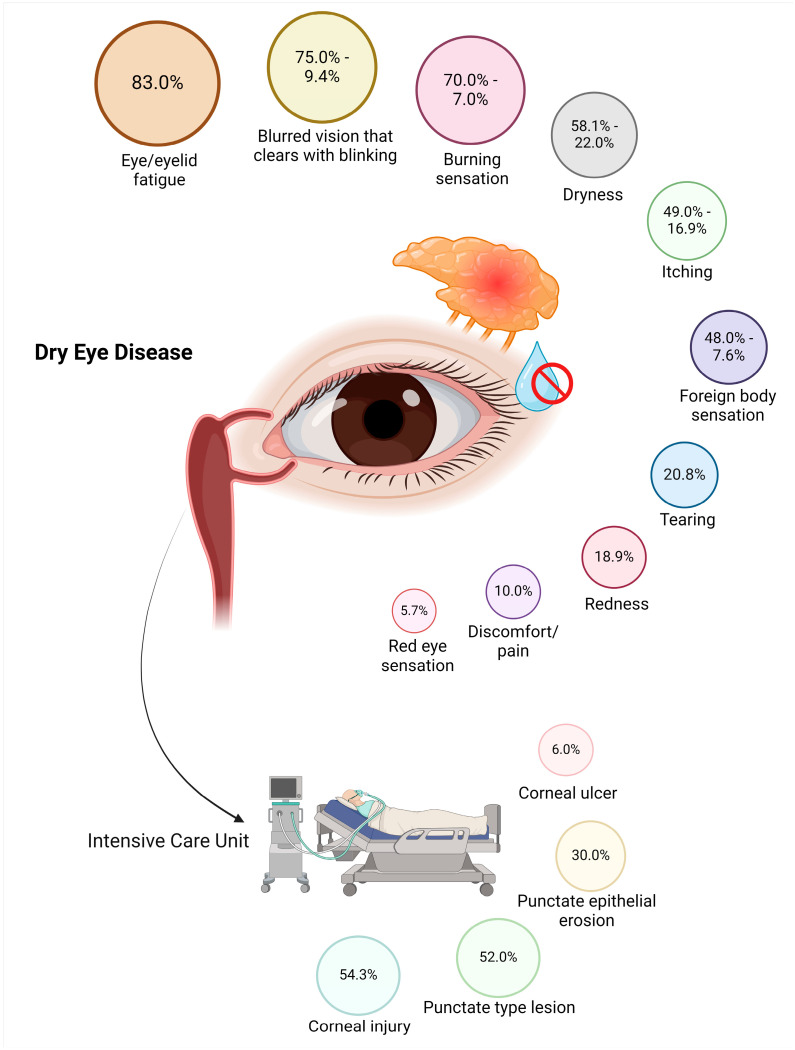
Symptoms and signs identified in patients with dry eye disease-related symptoms in South America. This figure was constructed using data from the studies of Dias D. et al., Solórzano-Fernández A. et al., Ulloa S. et al., Pérez C.M. and Alvarez D., and Vera F. et al. [19,20,22,23,25], and includes data from individuals in Brazil, Venezuela, and Ecuador. The arrow shows the percentage of dry eye disease symptoms among the Intensive Care Unit population.

Five studies examined the clinical presentation of dry eye disease-related symptoms in South American populations, identifying eye fatigue (83%), transient blurred vision (75.0%), burning sensation (52.0–70.0%), corneal injury (54.3%), Punctate lesions (52.0%), foreign body sensation (48.0%), itching (49%), and redness (18.9%) [19,20,22,23,25] (Table 2 and Figure 3).

Moreover, eleven studies examined factors associated with dry eye disease-related symptoms, identifying demographic factors like female sex [18,27,28,29,33], advanced age, particularly in women over 55 years [30], and in both sexes over 40 years [33]. Additionally, personal clinical histories, including connective tissue disorders, dyslipidemia, arterial hypertension, diabetes mellitus, thyroid disease, rheumatological diseases, benign prostatic hyperplasia [21,29,30,31,33], and the use of medications, including antidepressants, antiallergics, contraceptives, and isotretinoin, were also linked to DED symptoms [29,33] (Table 2).

Other additional associated factors included a history of eye surgery [31,33], contact lens use [18,29,33], use of eye drops [30], a history of keratoconus [18], and insufficient sleep (less than 7 or 6 h per night) [28,29]. In addition, there are often factors related to the use of electronic devices and screens, including prolonged screen time (more than 6 h per day) [18,25,28,29,33] and problematic use of the internet, found in men [26] (Table 2).

Also, according to the type of population, in the specific case of university students, including medical students, associated factors for DED symptoms included prolonged screen exposure (more than 6 h), previous diagnoses of dry eye, contact lens use, female sex, problematic internet use, sleeping less than 6 h, and the use of medications such as antidepressants and antihistamines [18,26,29]. Similarly, among administrative workers, prolonged use of computers (more than 6 h) was identified as a trait shared by this group with university students who are currently exposed to terminals with screens [25]. Meanwhile, in the general population, factors associated with DED included female sex, advanced age, comorbidities such as hypertension, dyslipidemia, connective tissue disorders, and the use of antidepressants. Additionally, the use of eye drops and a history of ocular surgery were considered associated factors [30,31,33]. Finally, in the population of intensive care unit (ICU) patients, related factors included corneal lesions and erosions, likely attributable to the quality of the ICU environment, including air quality and oxygen exposure [19].

## 4. Discussion

This review analyzed the available literature on the prevalence and associated factors of dry eye disease-related symptoms in South America, encompassing 16 studies from six countries. The reported prevalence of dry eye disease-related symptoms varied from 4% to 77.5%, with an average prevalence of 39.3%. This prevalence is higher than those reported in other systematic reviews globally that include findings from questionnaire symptoms and diagnostic clinical tests, including 18.5% in China [34], 8.1% in the United States [5], and 11.59% globally. However, it is lower than the 42.0% observed in Africa [35]. The substantial variability in prevalence may be due to differences in study populations, particularly the higher rates found among university students (58.6%) and administrative workers (57.9%). Additionally, the use of up to 10 different diagnostic questionnaires across the included studies likely contributed to this variability. Despite these differences, we believe that the findings of this review offer valuable insights into the reality of DED in the region. Notably, certain questionnaires, such as the OSDI and the Women’s Health Study questionnaire, have demonstrated significant diagnostic qualities, with the OSDI questionnaire showing a sensitivity of 0.6 and a specificity of 0.83 [36] and the Women’s Health Study questionnaire exhibiting a sensitivity of 0.77 and a specificity of 0.83 [37].

Sociodemographic, environmental, and medical factors play significant roles in the development of DED symptoms, which profoundly impact patients’ daily lives [15]. Female sex was identified as a major demographic factor associated with DED, a finding consistent with previous research from the United States, Korea, China, Spain, and Singapore [38,39,40,41,42,43,44,45]. This association is likely due to the influence of female hormones, particularly androgens, on tear secretion via the meibomian and lacrimal glands [34,46]. Advanced age was also shown to be an important factor in two studies included in this review [30,33], advanced age was also shown to be an important factor in two studies included in this review [44,47,48,49] that link age-related decline in tear gland function to DED [43,50,51]. While not explicitly identified in this review, environmental factors such as humidity and climate have been associated with DED in other regions [52,53,54,55], indicating the need for further research on these factors in South America.

The use of contact lenses was associated with dry eye disease-related symptoms in studies from Argentina, Brazil, and Chile [18,29,33], aligning with findings from prior research conducted in various contexts [4,15,39,42,50]. However, this association was not observed in more recent studies [45,47,56]. This contradiction could probably be explained because, as identified in this study, the association of the use of these devices with eye disease could be due to probable alterations in the tear film [57]. In contrast, the recent lack of association may be attributed to advances in the materials used in new contact lenses [58], suggesting a potential deficiency in access to these newer materials in the South American region.

Regarding the history of eye surgeries, this was identified as a significant associated factor for dry eye disease-related symptoms, particularly in populations from Brazil [29,31,33]. This finding aligns with previous studies showing that patients who undergo open-eye surgeries, such as cataract surgery, often experience high frequencies of alterations in ocular secretion [40,56,59,60,61].

Another common factor identified in this review was the use of or exposure to electronic devices or screens. This was especially evident in studies involving populations more frequently exposed to screens, such as administrative workers or university students [18,25,26,29]. This relationship has been widely supported by other research [15,47,49,50], with the effect attributed to increased tear evaporation due to reduced blinking. These findings are particularly relevant given the global trend towards increased use of electronic devices [62,63].

Similarly, personal habits such as insufficient sleep were associated with DED in studies conducted in Argentina and Brazil [28,29], consistent with previous research [45]. This association may be due to the positive effects of rapid eye movements during sleep on ocular moisture and lubrication, suggesting that insufficient sleep could compromise this protective mechanism [64].

The interaction of systemic diseases with DED was found to involve a wide range of conditions, including connective tissue disorders, dyslipidemia, arterial hypertension, diabetes mellitus, thyroid disease, rheumatological diseases, and benign prostatic hyperplasia. Some studies have established a link between hypertension and DED symptoms, while others have found no such connection [15]. Regarding thyroid disease, a systematic review with meta-analysis by Qian L. et al. identified an association between thyroid disorders and DED [65], a finding supported by recent research in the Saudi Arabian population [66]. However, Yu K. et al., in a study involving 535 adult patients with DED across twenty-seven care centers in the USA, found no association between thyroid disorders and DED signs [67]. While hypertension itself may not directly contribute to the development of DED, antihypertensive medications could play a role [68]. Rheumatoid diseases have also been associated with DED, as previously reported [39,69,70]. This connection may be due to increased inflammatory cytokines in the conjunctival epithelium, which can damage the tear glands [71,72].

Additionally, this review identified that certain medications, such as antidepressants, antihistamines, contraceptives, and isotretinoin, were associated with DED. Similar associations have been documented in other studies, particularly with antidepressants and antihistamines [73,74,75]. These associations may be explained by the potential effect of these drugs on muscarinic receptors in the conjunctival epithelium [75].

Furthermore, tobacco use was identified as a risk factor in the study by Yang I. et al. involving 2140 undergraduate students in Brazil [29]. Although this risk factor is not commonly reported, it has been previously identified in some populations, such as older adults or chronic smokers [75,76,77,78]. However, other studies have rejected this association [49,74,79].

On the other hand, various environmental factors have been implicated in the development of DED. Climatic conditions, such as high temperatures and low humidity, have been shown to exacerbate DED and contribute to more severe symptoms [80,81,82]. Additionally, altitude has been associated with a higher prevalence of dry eye in regions like Tibet and India, primarily due to decreased ambient humidity, increased exposure to UV rays, and higher wind exposure [54,83,84,85,86]. Although these environmental factors have not been specifically addressed in any of the studies included in this review, their consideration within the South American context is noteworthy. Several major cities, including Bogotá, Quito, and La Paz, are situated at altitudes exceeding 2500 m, areas that remain underexplored in DED research. Previous hypotheses suggest that these high-altitude locations may exhibit significant prevalence rates of dry eye disease.

Socioeconomic disparities and healthcare infrastructure significantly contribute to the variability in dry eye disease (DED) prevalence, as observed in this review [87,88]. Limited access to healthcare services in lower socioeconomic regions often results in underdiagnosis and undertreatment, skewing prevalence rates. In contrast, regions with robust healthcare systems typically offer better management of eye conditions, which may lead to lower rates of DED through effective interventions and public health initiatives, as seen in Brazil. However, the heterogeneity in dry eye disease-related symptoms must also be evaluated in light of additional factors. Occupational exposures common in lower-income areas may exacerbate DED symptoms, while lifestyle factors, such as increased screen time among urban workers, are associated with higher prevalence rates of DED.

The findings of this study highlight the wide distribution of dry eye disease-related symptoms in South America and underscore its importance as a public health problem in the region. These findings are particularly significant given that, although DED has been extensively studied in other settings, data from South American populations have often not been represented in systematic reviews [15,65]. This review also emphasizes the need for more solid and robust epidemiological studies employing clinical diagnostic criteria that would more accurately identify the prevalence of DED throughout the region, particularly in countries where data are lacking, such as Colombia, Bolivia, Paraguay, and Uruguay.

### Limitations

This research has several limitations that warrant acknowledgment. Firstly, the focus on studies published solely in English and Spanish may have excluded relevant research published in other languages prevalent in the region, such as Portuguese, potentially limiting the comprehensiveness of the review. Secondly, the absence of standardization in the diagnostic methods across the included studies—evidenced by substantial variability in the tools used, such as differing questionnaires and criteria for diagnosing dry eye disease introduces inconsistencies in the prevalence estimates for DED symptoms, making direct comparisons challenging.

Moreover, the predominant reliance on cross-sectional study designs in most of the included studies restricts the ability to establish causal relationships among factors associated with dry eye disease symptoms. Additionally, the diversity in the population groups studied—ranging from university students to the general population and intensive care unit patients—combined with the lack of data from several South American countries may introduce biases and affect the generalizability of the findings.

Furthermore, although this review aimed to encompass a broad spectrum of associated factors, there may be unexamined variables, including environmental influences, that could further clarify the etiology of DED. These limitations highlight the need for future research to address these gaps, employing standardized methodologies and diverse populations to enhance our understanding of DED in South America.

## 5. Conclusions

The prevalence of dry eye disease-related symptoms in South America is significantly higher than global averages, highlighting the unique challenges faced by populations in this region. Several key factors contributing to this elevated prevalence were identified, including demographic characteristics such as female sex and older age, environmental factors like prolonged screen time and inadequate sleep, and medical conditions such as connective tissue disorders, hypertension, and the use of certain medications, particularly antihypertensives, antidepressants, and antihistamines.

This study highlights the critical need for more comprehensive data collection, especially from underrepresented South American countries, and underscores the importance of conducting larger epidemiological studies across the continent. These efforts are essential to fully understand the burden of dry eye disease and to develop effective public health strategies tailored to the specific needs of these populations.

## Figures and Tables

**Table 1:** Characteristics of the studies on prevalence and associated factors of dry eye disease-related symptoms in South America included in this systematic review.

Author, Year	Country (Region/City)	Study Design	Number of Participants	Population
Cartes C. et al., 2021 [18]	Chile (Santiado de Chile)	Cross-sectional	1450	University students receiving online classes
Dias D. et al., 2016 [19]	Brazil (Belo Horizonte)	Cohort concurrent	258	Intensive care unit patients
Solórzano-Fernández A. et al., 2023 [20]	Ecuador (Manabi)	Cross-sectional	365	Administrative workers
Arcentales P. et al., 2020 [21]	Ecuador (Manabi)	Case-control study	1908	Patients diagnosed with dry eye and their undiagnosed family members
Ulloa S. et al., 2020 [22]	Ecuador (Guayaquil)	Cross-sectional	60	Company workers
Pérez C.M. and Alvarez D., 2001 [23]	Venezuela (Caracas)	Cross-sectional	89	Patients with rheumatoid arthritis in the Ophthalmology Service
Holgado M. et al., 2023 [24]	Argentina (Cordoba)	Cross-sectional and prospective	100	Ophthalmology patients between 20 and 50 years of age
Vera F. et al., 2022 [25]	Ecuador (Guayaquil)	Cross-sectional	100	Administrative workers
Condori-Meza I. et al., 2021 [26]	Peru	Cross-sectional study	844	Medical students
Goya M., 2023 [27]	Chile (Santiago de Chile)	Cross-sectional study	205	Dry eye disease patients
Torres R. et al., 2023 [28]	Argentina	Cross-sectional	10,812	General population older than 12 years
Yang I., 2021 [29]	Brazil	Cross-sectional	2140	Undergraduate students
Guedes C.L. et al., 2022 [30]	Brazil (Sao Paulo)	Cross-sectional	582	General population
Pereira L. et al., 2017 [31]	Brazil (Cássia dos Coqueiros)	Cross-sectional	200	General population
Mitchell J. et al., 2008 [32]	Venezuela	Cross-sectional	1261	General population
de Castro J. et al., 2018 [33]	Brazil	Cross-sectional	3107	General population

**Table 2:** Systematic summary of the prevalence and factors associated with dry eye disease-related symptoms in South America.

Author, Year	Country (Region/City)	Diagnosis Method	Prevalence of Symptoms Related to DED	Etiology	Clinical Presentation	Associated Factors
Cartes C. et al., 2021 [18]	Chile (Santiago de Chile)	Dry Eye Questionnaire (DEQ-5)	77.5%	Computer vision syndrome	N/A	Female sex, previous dry eye diagnosis, keratoconus, contact lens use, and duration of visual display terminal use.
Dias D. et al., 2016 [19]	Brazil (Belo Horizonte)	Schirmer I test	53.0%	Dry eye disease	Corneal lesion (54.3%), Punctate type lesion (52.0%), and Punctate epithelial erosions (30.0%)	O2 in ambient air, O2 by nasal catheter, oral diet released, and enteral diet route.
Solórzano-Fernández A. et al., 2023 [20]	Ecuador (Manabi)	McMonnies questionnaire	49.0%	Computer vision syndrome	Itching (49.0%), dryness (22.0%), burning (7%), and discomfort/pain (10%)	N/A
Arcentales P. et al., 2020 [21]	Ecuador (Manabi)	Ocular Surface Disease Index (OSDI) questionnaire	33.3%	Dry eye disease	N/A	Hypertension, diabetes, hepatitis C, thyroid disease, connective tissue disease, benign prostatic hyperplasia, rosacea, depression, post-traumatic stress disorder, and hematopoietic stem cell transplantation; neck radiation therapy and estrogen therapy after menopause.
Ulloa S. et al., 2020 [22]	Ecuador (Guayaquil)	BUT test, Schirmer II test, and Ferning test	76.7%	Dry eye disease	Redness (18.87%), burning 16.98%, itching (16.98%), blurred vision that clears with blinking (9.43%), tearing (20.75%), redness (5.66%), foreign body sensation (7.55%)	N/A
Pérez C.M. and Alvarez D., 2001 [23]	Venezuela (Caracas)	Schirmer’s test and Vital’s staining test	37.1%	Dry eye disease	Burning (52.0%), foreign body sensation (48.0%), and dryness (58.1%)	N/A
Holgado M. et al., 2023 [24]	Argentina (Cordoba)	Ocular Surface Disease Index (OSDI) questionnaire, fluorescence staining, lissamine green staining, and precorneal breakup time	22.0% by OSDI, 26% by fluorescence staining, 46% by lissamine green staining, and 52% by precorneal breakup time	Computer vision syndrome	N/A	N/A
Vera F. et al., 2022 [25]	Ecuador (Guayaquil)	Donate questionnaire	48.0%	Computer vision syndrome	Eye/eyelid fatigue (83.0%), blurred vision that clears with blinking (75.0%), and burning (70.0%)	Computer use more than 6 h per day and more than 6 years working in the same position
Condori-Meza I. et al., 2021 [26]	Peru	Ocular Surface Disease Index (OSDI) questionnaire	70.9%	Computer vision syndrome	N/A	Problematic internet use in men.
Goya M.C., 2023 [27]	Chile (Santiago de Chile)	DEWS II criteria	100.0%	Dry eye disease	N/A	Female sex
Torres R. et al., 2023 [28]	Argentina	Women’s Health Study (WHS) dry eye questionnaire	42.1%	Dry eye disease	N/A	Female sex, use of digital screens for more than 6 h per day, and sleeping less than 7 h per day
Yang I., et al., 2021 [29]	Brazil	Ocular Surface Disease Index (OSDI) and the Women’s Health Study (WHS) questionnaires	34.4% from the OSDI and 23.5% from the WHS	Dry eye disease	N/A	Both questionnaires: female sex, screen use of more than 6 h per day, anti-allergy medication, and rheumatological disease. From the WHS questionnaire: ocular surgery, contact lens wear, and isotretinoin use. From the OSDI questionnaire: contraceptive use, antidepressant use, smoking, and less than 6 h of sleep.
Guedes C.L. et al., 2022 [30]	Brazil (Sao Paulo)	Short dry eye questionnaire	24.4%	Dry eye disease	N/A	In women: age older than 55 years, hypertension, and eye drops use. In men: eye drops use.
Pereira L. et al., 2017 [31]	Brazil (Cássia dos Coqueiros)	Ocular Surface Disease Index (OSDI) score > 20, or Schirmer’s test (ST) < 10 mm, or tear film breakup time (TFBUT) ≤ 6 s	19.4%	Dry eye disease	N/A	Menopause, eye surgery, and dyslipidemia.
Mitchell J. et al., 2008 [32]	Venezuela	N/A	4.0%	Dry eye disease	N/A	N/A
de Castro J. et al., 2018 [33]	Brazil	Short dry eye questionnaire	12.8%	Dry eye disease	N/A	Female sex, older than 40 years, history of ocular surgery, connective tissue disorders, contact lens wear, computer use > 6 h per day, antidepressant use, anti-allergy drug, and menopause.

## Data Availability

Not applicable.
